# Supplementary material for: The genome sequence of Plagiothecium denticulatum (Hedw.) Schimp. (Hypnales: Plagiotheciaceae)
Source: Wellcome Open Res. 2026 Jul 7;11:424. [Version 1] doi: 10.12688/wellcomeopenres.26968.1 (PMC13594601; doi:10.12688/wellcomeopenres.26968.1)
Supplement: Peer review history

# Reviewer report: Reviewer response for version 1

**DOI:** 10.21956/wellcomeopenres.29689.r166348

**Published:** 2026-09-21

**Authors:** Florent Sylvestre

**Affiliations:** 1 University Laval, Québec, Canada

Dear editor,

 The work of D.Bell and D.G Long provide a high-quality reference genome for *Pleiochasium denticulatum*. Relying on HiFi long read sequencing for initial genome assembly and Hi-C data for contigs scaffolding, the assembly workflow follows state-of-the-art standards and will represent a great resource for future projects. Results include expected metrics and confirm the quality of this genome assembly, with expected chromosome counts and genome size.

 While expected genome size is estimated by the authors using flow cytometry, authors could elaborate more on previous research. In the introduction, they redirect the readers toward existing literature ( **“The chromosomally complete genome presented here is consistent with most published chromosome counts (** **Fritsch, 1991** **) and genome size estimates ( Bainard *et al.*, 2020** **) for the species. »**), but I think that explicitly reporting that information would make the paper easier to follow.

  The methods are mostly clearly written and reproducible, and meet the recent literature on genome assembly. However, their use of the Assembly Screen for Cobionts and Contaminants pipeline for decontamination could be more explicit, as this pipeline incorporates many tools and it is not clear whether they used all or part of these tools.

 Other than these two small suggestions, this work provides a comprehensive report of a high-quality genome assembly which I fully recommend.

Are sufficient details of methods and materials provided to allow replication by others?

Partly

Is the rationale for creating the dataset(s) clearly described?

Yes

Are the datasets clearly presented in a useable and accessible format?

Yes

Are the protocols appropriate and is the work technically sound?

Yes

Reviewer Expertise:

Evolutionary genomic, genome assembly and population genetics

I confirm that I have read this submission and believe that I have an appropriate level of expertise to confirm that it is of an acceptable scientific standard.

# Reviewer report: Reviewer response for version 1

**DOI:** 10.21956/wellcomeopenres.29689.r162513

**Published:** 2026-08-13

**Authors:** Ian M Carr

**Affiliations:** 1 University of Leeds, Leeds, UK

The paper is a standard Tree of Life genome submission paper, and so all methods and results are covered.

 The only issues are:

 Figure 3 has a grey vertical cross and I've no idea what it is and the paper doesn't explain its presence.Either remove it or explain what it is.

 Figure 4 is a graph with two cures (one black, one red). The figure legend describes the black curve and then says "the remaining curves" but there is only one other curve so why not say "the red curve". Either change "the remaining curves" to "the remaining curve" or "the red curve".

Are sufficient details of methods and materials provided to allow replication by others?

Yes

Is the rationale for creating the dataset(s) clearly described?

Yes

Are the datasets clearly presented in a useable and accessible format?

Yes

Are the protocols appropriate and is the work technically sound?

Yes

Reviewer Expertise:

NGS data, software development eDNA, genome assembly and disease gene detection.

I confirm that I have read this submission and believe that I have an appropriate level of expertise to confirm that it is of an acceptable scientific standard.
